# Downsizing Mo_6_I_12_ to Nanocrystals Unveils Visible-Light Photocatalytic Antibacterial Activity

**DOI:** 10.1021/acs.inorgchem.5c05876

**Published:** 2026-03-18

**Authors:** Michaela Kubáňová, Martin Št’astný, Eric Bourhis, Petr Bezdička, Jakub Tolasz, Aimin Yao, Jean-François Halet, Mouna Ben Yahia, Régis Gautier, Jaroslav Zelenka, Kamil Lang, Régis Guégan, Kaplan Kirakci

**Affiliations:** † Department of Biochemistry and Microbiology, 52735 University of Chemistry and Technology Prague, 166 28 Praha, Czech Republic; ‡ Institute of Inorganic Chemistry of the Czech Academy of Sciences, 250 68 Husinec-Řež, Czech Republic; § Interfaces, Confinement, Matériaux et Nanostructures ICMN-UMR 7374, 129631 CNRS-Université d’Orléans, 1 Rue de la Férollerie, 45100 Orléans, France; ∥ Univ Rennes, École Nationale Supérieure de Chimie de Rennes, CNRS, ISCR UMR 6226, 35000 Rennes, France; ⊥ CNRS - Saint-Gobain - NIMS, IRL 3629, Laboratory for Innovative Key Materials and Structures (LINK), 1-1 Namiki, Tsukuba 305-0044, Japan; # Institute Charles Gerhardt of Montpellier, 27037 Univ. Montpellier, CNRS, ENSCM, 34293 Montpellier, France

## Abstract

Molybdenum­(II) iodide (Mo_6_I_12_) serves as a versatile precursor to phosphorescent octahedral molybdenum cluster complexes that exhibit properties relevant to energy, environmental, and biomedical applications. Despite its straightforward synthesis, stability, and extended visible-light absorption, the photophysical and photocatalytic properties of Mo_6_I_12_ have remained unexplored, because the compound has historically been available only as an insoluble bulk material. Herein, we report the top-down preparation of Mo_6_I_12_ nanocrystals via ultrasonic treatment, yielding stable colloidal suspensions in acetone and water. The nanocrystals exhibited broad visible-light absorption extending up to ∼700 nm and weak red to near-infrared photoluminescence intensifying at low temperatures. These features indicate an indirect semiconducting nature, which was confirmed by density functional calculations. Upon blue-light illumination, the nanocrystals generate reactive oxygen species, including the hydroxyl radical via a photocatalytic mechanism that operates even under anaerobic conditions. Their photocatalytic potential in the context of water disinfection was validated through bacterial photodynamic inactivation, demonstrating effective inactivation (up to >4 log reduction) of clinically relevant strains of Gram-positive bacteria under visible-light irradiation. The low toxicity of the nanocrystals on HeLa cells highlighted their favorable safety profile for water disinfection. This work reveals the unique light-induced properties of Mo_6_I_12_ nanocrystals and establishes them as promising materials for photocatalytic and photodynamic applications.

## Graphical abstract

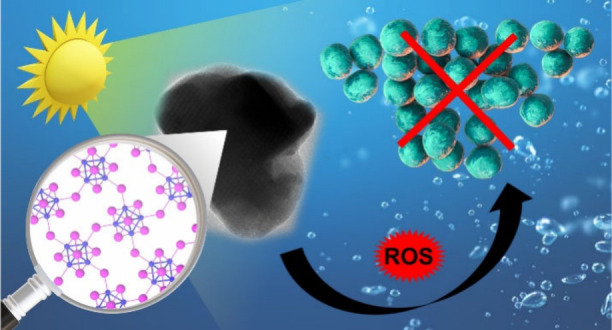

## Introduction

Molybdenum­(II) iodide (Mo_6_I_12_) is a black, crystalline solid. It is readily obtained by reaction between molybdenum and iodine elements at high temperatures.^1^ It is stable in air and insoluble in common organic solvents, which is consistent with its strong internal bonding and cohesive structure.^2^ The compound, isostructural to Mo_6_Cl_12_ and Mo_6_Br_12_, features octahedral Mo_6_ clusters, where six molybdenum atoms are interconnected through Mo–Mo metallic bonds. Each Mo_6_ cluster is surrounded by eight face-capping inner iodides (I^i^) that contribute to the cluster’s integrity and six labile apical iodides (I^a^). Four of the apical iodides bridge adjacent clusters forming monolayers, which are stacked via van der Waals interactions, reminiscent of the molybdenum disulfide’s structure (Figure 1). Mo_6_I_12_ or [Mo_6_I^i^_8_I^a^_2_(I^a^_4/2_)], according to its developed formula, is indeed the precursor used to synthesize the whole family of [Mo_6_I^i^_8_L^a^_6_]*^n^* molecular complexes (L is a two-electron donor ligand, and *n* is the cluster charge), which possess remarkable photophysical properties relevant for photonic, environmental, and health applications.^3–8^

**Figure 1:**
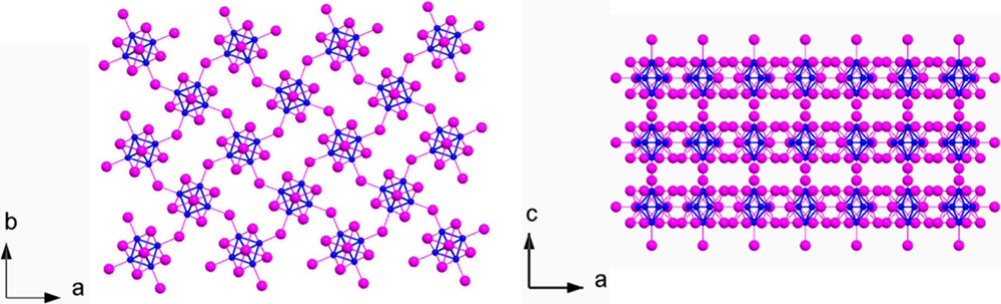
Crystallographic representation of the structure of Mo_6_I_12_ (COD ID 1529547):^9^ (left) projection along the *c* axis of one Mo_6_I_12_ layer and (right) projection along the *b* axis of three stacked Mo_6_I_12_ layers. Color code: blue for Mo and magenta for I.

It turns out that little is known about the photophysical properties of Mo_6_I_12_, which is assumed to be a narrow-band gap semiconductor due to its valence electron count of 24 corresponding to a fulfilled valence band.^10,11^ Finding efficient ways to reach nanosized Mo_6_I_12_ could allow for the study of its photophysical properties and open new avenues for application in various fields, from photocatalysis to therapeutic applications. Indeed, molybdenum plays a crucial role in enhancing photocatalytic microbial activity, a process that exploits light-activated photosensitizers and/or photocatalysts to kill or inactivate microorganisms and has emerged as a potent alternative to the use of traditional antibacterial agents. For instance, MoS_2_, which shows a lamellar structure reminiscent of that of MoI_2_, has shown visible-light bacterial photoinactivation properties.^12^ Notably, several stand-alone Mo_6_ cluster complexes or Mo_6_-derived materials have shown efficient activity for blue-light photoinactivation of planktonic cultures^13–15^ or photoinhibition of biofilms.^16–18^

Among the methods to reduce bulk material to nanometric scale, ultrasonication has been demonstrated as an efficient top-down approach. It involves the application of high-frequency ultrasonic waves to a liquid medium. These sound waves generate alternating high- and low-pressure cycles, leading to the formation and collapse of microbubbles, a process known as cavitation. The intense localized energy released during cavitation facilitates nanoparticle synthesis by breaking down larger particles, promoting nucleation, and enhancing mixing and dispersion. This method is efficient and scalable and can be used for a variety of materials, offering control over particle size and distribution.^19–22^

Herein, we demonstrate the top-down preparation of Mo_6_I_12_ nanocrystals by ultrasonic treatment and their use in bacterial photoinactivation. The nanocrystals were characterized by powder X-ray diffraction, electron microscopy, Raman spectroscopy, and X-ray photoelectron spectroscopy analysis. Dispersions of the nanocrystals in acetone and deionized water were characterized by dynamic light scattering. Aqueous dispersions and solid samples were measured by luminescence spectroscopy. The photophysical and spectroscopic measurements were further analyzed in light of density functional theory calculations. The blue-light-induced antibacterial activity of the nanocrystals was studied on planktonic cultures of *Enterococcus faecalis*, *Staphylococcus aureus*, and *Escherichia coli*.

## Results and Discussion

### Characterizations

Nanocrystals of Mo_6_I_12_ (**nMo**_**6**_ **I**_**12**_) were obtained by ultrasonic treatment of bulk Mo_6_I_12_ in *N*-methyl-2-pyrrolidone (NMP) using a 2 kW ultrasound water-cooled reactor for 2 h followed by several cycles of centrifugation and washing with acetone to remove traces of NMP (see the Experimental Section for details). The powder X-ray diffraction (XRD) pattern of **nMo**_**6**_ **I**_**12**_ matched the reported crystal structure of Mo_6_I_12_ and indicated an average crystallite size of 108 nm, slightly smaller than that of bulk Mo_6_I_12_ with a 138 nm average crystallite size, as obtained by the Scherrer equation (Figure 2A, Figure S1, and Table S1).

**Figure 2:**
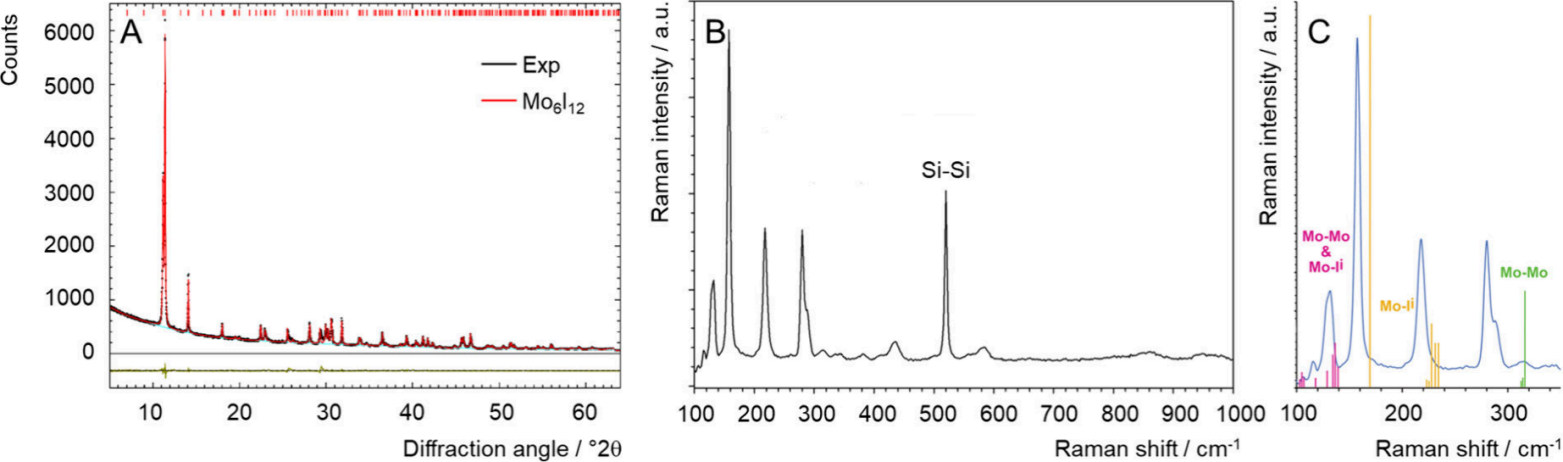
(A) Powder X-ray diffraction patterns of **nMo**_**6**_ **I**_**12**_ with the corresponding Rietveld fit using the reported crystal structure of Mo_6_I_12_ (COD ID 1529547). Residuals are shown at the bottom of the patterns.^9^ (B) Raman spectrum of **nMo**_**6**_ **I**_**12**_ deposited on a silicon substrate. (C) Close-up of the experimental Raman spectrum (blue line) together with the main contributions computed from DFT calculations. The color of the computed Raman bands indicates its vibrational origin.

The Raman analysis of a **nMo**_**6**_ **I**_**12**_ thin film deposited on a Si substrate displayed well-defined intense and sharp peaks, as shown in Figure 2B. In order to better understand the origin of each Raman shift, a coupled-perturbed-Hartree–Fock/Kohn–Sham scheme was used to compute the Raman spectrum of Mo_6_I_12_.^23,24^ Most intense Raman modes and their respective intensities are reported in Table S2. The most significant computed contributions to the Raman spectrum of bulk Mo_6_I_12_ are sketched together with the experimental spectrum in Figure 2C. It is worth noting that the experimental and calculated Raman spectra show excellent overall qualitative agreement, particularly in the relative intensities of the peaks. The discrepancies between the experimental and computed frequencies tend to increase at higher frequency, which can arise from computational factors, such as the choice of the exchange-correlation functional. It is remarkable that our computational and measured Raman peaks appear at frequencies very similar to those of individual molecular clusters (Bu_4_N)_2_[Mo_6_I_14_] constituting the layers.^25^

In agreement with density functional theory (DFT) calculations, the main Raman peaks are observed between 100 and 400 cm^–1^, coming from the bonds constituting the Mo cluster: Mo–I^a^, Mo–I^i^, and Mo–Mo vibrations. The I^i^ ligands cap the triangular faces of the metallic octahedral, whereas the I^a^ ligands are connected to only one Mo atom per octahedral unit. Some of the latter Raman shifts can be shared with neighboring cluster units. The shifts lying at ca. 105, 115, and 130 cm^–1^ are assigned to Mo–Mo and Mo–I^i^ vibrations, whereas the shifts at ca. 160 and 220 cm^–1^ can be assigned to Mo–I^i^ vibrations. The peak located at 315 cm^–1^ corresponds to Mo–Mo vibrations. The Raman shift, observed at a higher frequency, around 520 cm^–1^, does not arise from the cluster; it is a typical vibration of Si atoms within the crystalline lattice of the Si substrate used for the preparation of the samples for both Raman and XPS characterizations.

Based on the results presented above, ultrasonic treatment for 2 h does not lead to measurable reorganization of the cluster units within the layered structure or affect its crystallinity. Transmission (TEM) and scanning (SEM) electron microcopy of **nMo**_**6**_ **I**_**12**_ revealed nanocrystals with a mean size of 145 ± 62 nm (Figure 3), comparable to that of the crystalline domains obtained by powder XRD (Figure 2). Energy-dispersive X-ray spectroscopy (TEM-EDS) evidenced a Mo:I molar ratio of 0.49 ± 0.04, which is consistent with the title formula and suggests that no extensive substitution of the iodine ligands occurred during the ultrasonic treatment.

**Figure 3:**
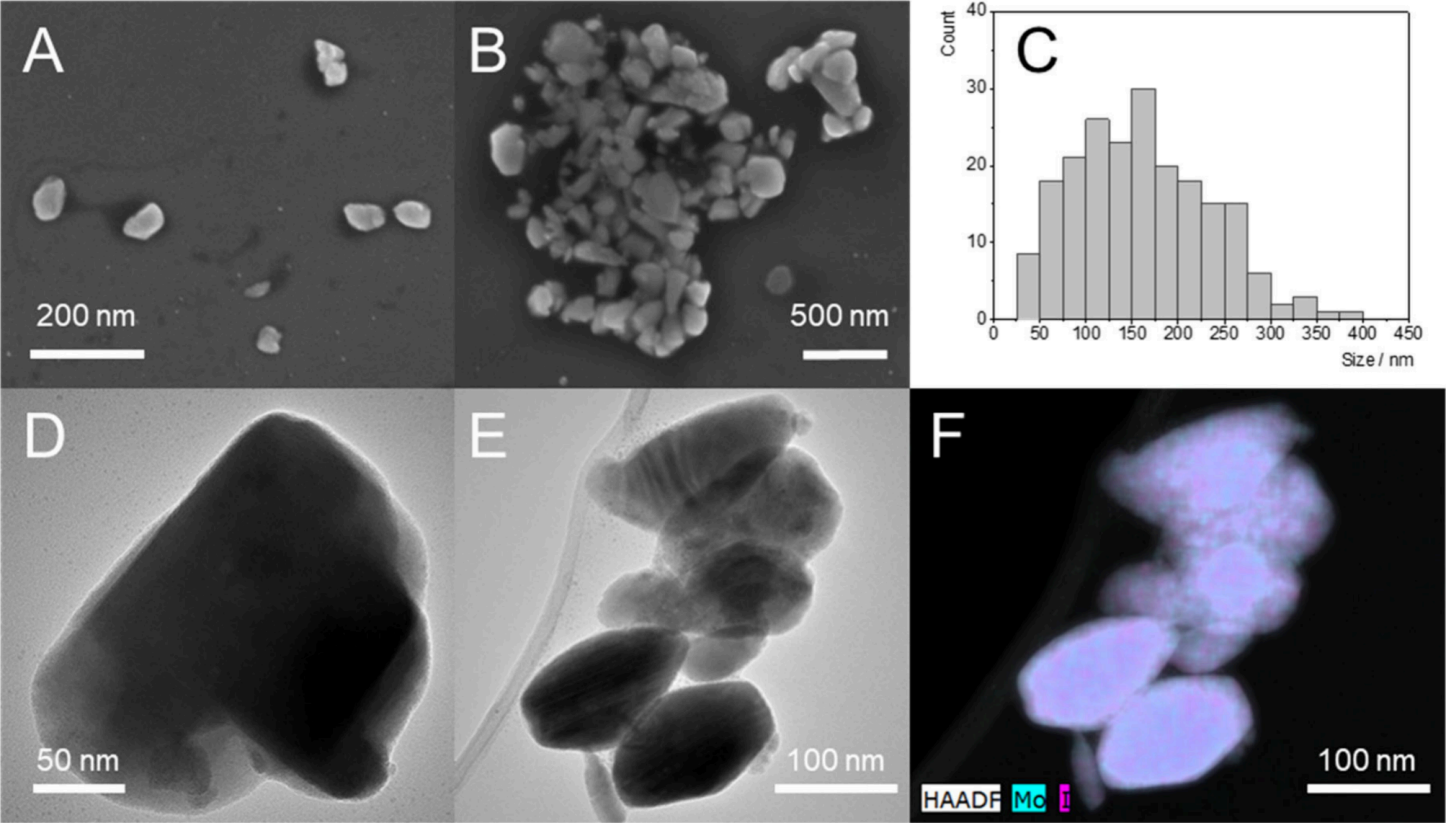
(A and B) SEM and (D and E) TEM images of **nMo**_**6**_ **I**_**12**_ deposited by drop casting of an acetone dispersion of the nanoparticles. (C) Size distribution obtained from SEM images. (F) TEM-EDS elemental mapping of Mo and I.

X-ray photoelectron spectroscopy (XPS) was performed on **nMo**_**6**_ **I**_**12**_ to obtain a fine analysis of the chemical states of the elements. **nMo**_**6**_ **I**_**12**_ was deposited on a Si substrate as a thin film, and the XPS spectrum was collected within a few nanometers range in depth (Figure 4A, Figure S2, and Table S3). In addition to Mo and I, the sample contained a significant amount of C and O; their sources may be adventitious carbon and the use of acetone and NMP solvent. The analysis of the integrated intensity evidenced a Mo:I ratio of 0.52, in agreement with that obtained by the TEM-EDS technique. The Mo 3d region was described well by two main Mo 3d_5/2–3/2_ peaks with binding energies of 228.9–232.0 eV, consistent with a Mo­(II) oxidation state (Figure 4B). The I 3d region exhibited a superposition of two main doublets, I 3d_5/2–3/2_ at binding energies of 619.1–630.6 and 620.5–632.0 eV, reflecting the respective coordination strength of apical and inner iodides in **nMo**_**6**_ **I**_**12**_ (Figure 4C). The analysis of the integrated intensity of these two I 3d_5/2–3/2_ peaks matched the title formula [Mo_6_I^i^_8_I^a^_2_(I^a^_4/2_)] with a ratio between apical iodides I^a^ and inner iodides I^i^ of 0.51.

**Figure 4:**
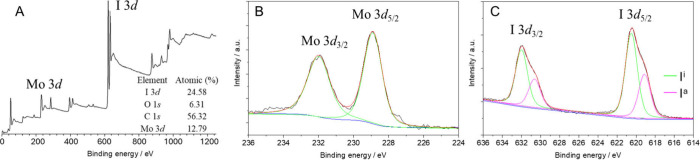
(A) XPS spectra of **nMo**_**6**_ **I**_**12**_ and corresponding fits of (B) Mo 3d and (C) I 3d core level signals.

The mean size by number of a colloidal dispersion of **nMo**_**6**_ **I**_**12**_ in acetone measured by dynamic light scattering was 155 ± 66 nm (hydrodynamic diameter (*Z*-average) of 217 nm, polydispersity index (PDI) of 0.14) (Figure 5A and Figure S3, and Table S4), comparable to the size obtained by SEM and TEM. A mean size by number of 178 ± 70 nm (*Z*-average of 252 nm, PDI of 0.20) measured in aqueous dispersions suggests limited aggregation of particles in this solvent. Electrophoretic light scattering evidenced a ζ potential of −22 ± 12 mV for **nMo**_**6**_ **I**_**12**_ in water, consistent with the presence of unshared apical ligands at the surface of the nanocrystals, providing a negative charge (Figure 5B).

**Figure 5:**
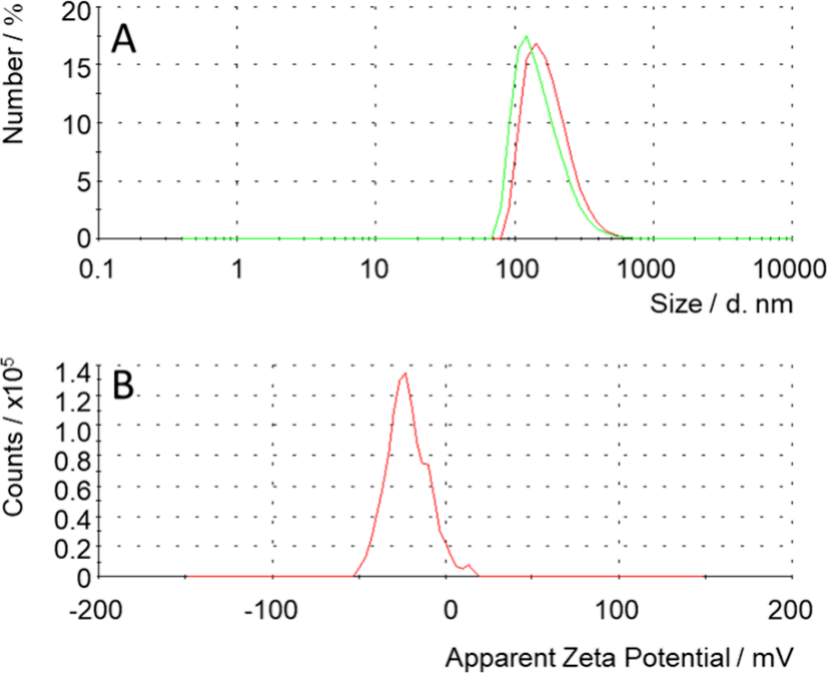
(A) Size distribution by number of acetone (green) and water (red) dispersions of **nMo**_**6**_ **I**_**12**_, as obtained by dynamic light scattering. (B) ζ potential distribution of a dispersion of **nMo**_**6**_ **I**_**12**_ in deionized water (pH ∼6), as obtained by electrophoretic light scattering.

Overall, the ultrasonication of bulk Mo_6_I_12_ leads to nanocrystals with preserved crystallinity and a preserved chemical composition. The slightly smaller size of crystalline domains suggests that ultrasonication in NMP results in the dissolution of grain boundaries between crystalline domains leading to colloidally stable nanocrystals.

### Photophysical Studies

The photophysical properties of **nMo**_**6**_ **I**_**12**_ were first studied in deionized water (Figure 6 and Table 1). Aqueous dispersions of **nMo**_**6**_ **I**_**12**_ displayed broad absorption bands with maxima at approximately 450 and 600 nm, and an onset at 700 nm, corresponding to a band gap (*E*_g_) of 1.78 eV, according to the Tauc plot for indirect semiconductors (Figures S4 and S5). The absorption is extended to the visible region when compared to (Bu_4_N)_2_[Mo_6_I_14_], which shows an onset at 600 nm in acetonitrile.^4^ Upon excitation at 450 nm, a weak red photoluminescence was observed, with a maximum at approximately 675 nm. It was possible to discern a secondary emission in the form of a very broad tail in the 800–900 nm spectral region. Both excitation spectra recorded at 680 and 850 nm resemble the corresponding absorption spectra, suggesting that the observed emissions originate from photoexcitation of **nMo**_**6**_ **I**_**12**_ (Figure S4). The luminescence quantum yield in an argon-saturated aqueous dispersion was very low (<0.01), and the emission lifetimes were remarkably short, 20 and 490 ns at 680 and 850 nm, respectively, indicating severe restriction of radiative relaxation processes. Saturation of the aqueous dispersion with air did not significantly alter the emission decay kinetics (Figure 6). Luminescent Mo_6_ clusters generally show high phosphorescence quantum yields and display a lifetime in the tens or hundreds of microseconds in the absence of oxygen.^4^ Thus, the photoluminescence of **nMo**_**6**_ **I**_**12**_ shows spectral features comparable to those of molecular Mo_6_ complexes; however, the emission quantum efficiency is much lower, and the decay kinetics are comparably faster.

**Table 1:** Photophysical Properties of the Water Dispersion and Solid Sample of **Mo**_**6**_ **I**_**12**_ ^a^

sample	temperature (K)	λ_L_ (nm)	Φ_L_	τ_L_ (ns)
water	300	675	<0.01	20^b^ (19^d^), 490^c^ (470^e^)
solid	300	684	<0.01	17,^f^ 360^g^
	80	855	0.08	11 200^g^

a Abbreviations: λ_L_, luminescence maximum (λ_exc_ = 450 nm); τ_L_, amplitude average lifetime (λ_exc_ = 405 nm); Φ_L_, luminescence quantum yield (λ_exc_ = 450 nm; the experimental error of Φ_L_ is ±0.01).

b Recorded at 680 nm in argon-saturated aqueous dispersions.

c Recorded at 850 nm in argon-saturated aqueous dispersions.

d Recorded at 680 nm in air-saturated aqueous dispersions.

e Recorded at 850 nm in air-saturated aqueous dispersions.

f Recorded at 680 nm in air atmosphere.

g Recorded at 850 nm in air atmosphere.

**Figure 6:**
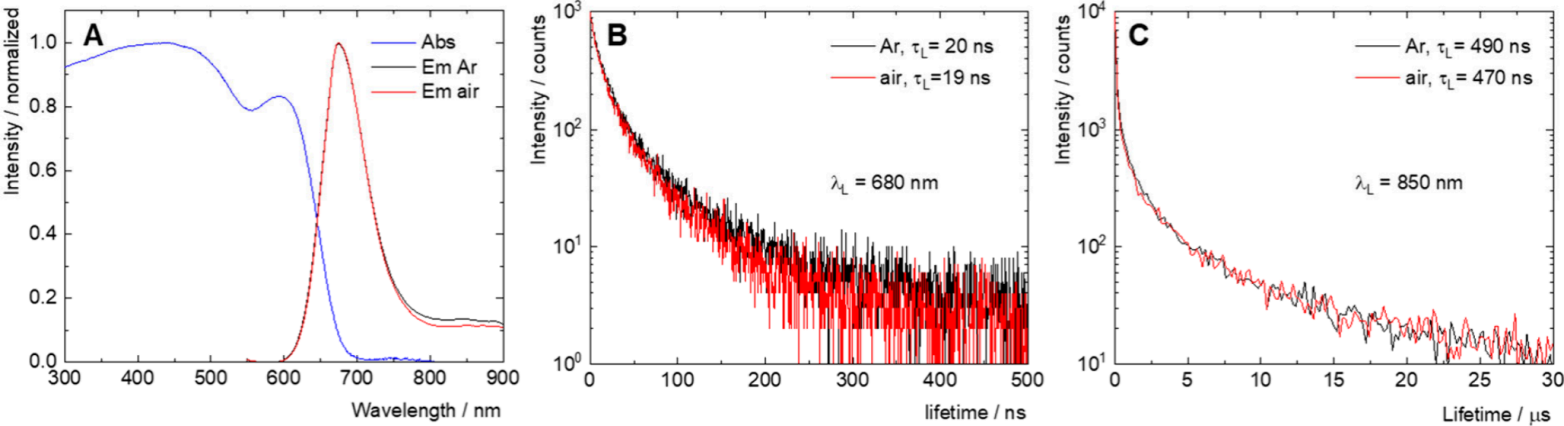
(A) Normalized absorption spectrum (Abs) and emission spectra of air-saturated (Em air) and argon-saturated (Em Ar) water dispersions of **nMo**_**6**_ **I**_**12**_, excited at 450 nm. Luminescence decay kinetics of air- and argon-saturated water dispersions of **nMo**_**6**_ **I**_**12**_, excited at 405 nm, were recorded at (B) 680 and (C) 850 nm.

In order to identify the origin of the observed photoluminescence, the photophysical properties of a solid sample of **nMo**_**6**_ **I**_**12**_ were studied in the range of 80–300 K (Figure 7). The emission spectrum, Φ_L_, and emission lifetime were similar to those of water dispersions at room temperature, albeit a red-shift of the maximum to 684 nm was observed. Upon cooling, the luminescence in the NIR was enhanced, gradually overcoming the red emission and leading to a broad band with a maximum at 855 nm at 80 K. The lifetime of this emission dramatically increased from 360 ns at 300 K to 11.2 μs at 80 K. Similarly, Φ_L_, which could not be measured at room temperature, increased to 8% at 80 K. Meanwhile, the red luminescence experienced a blue-shift of its maximum to 640 nm at 80 K and an increase in the lifetime to 217 ns at 160 K. Note that the lifetime of the red emission was not measurable at lower temperature due to strong overlap with the predominant emission band in the NIR. The excitation spectra recorded at 680 and 850 nm displayed blue-shifts and a steepening of the onset upon cooling. Because Mo_6_-based compounds were previously reported to be good scintillators, the radioluminescence of solid **nMo**_**6**_ **I**_**12**_ upon irradiation with an X-ray source (60 keV, 200 mA) was also recorded (Figure S6).^26^ The radioluminescence spectra matched that obtained by excitation at 450 nm, suggesting that photoinduced relaxation and radioinduced relaxation of the excited states are comparable.

**Figure 7:**
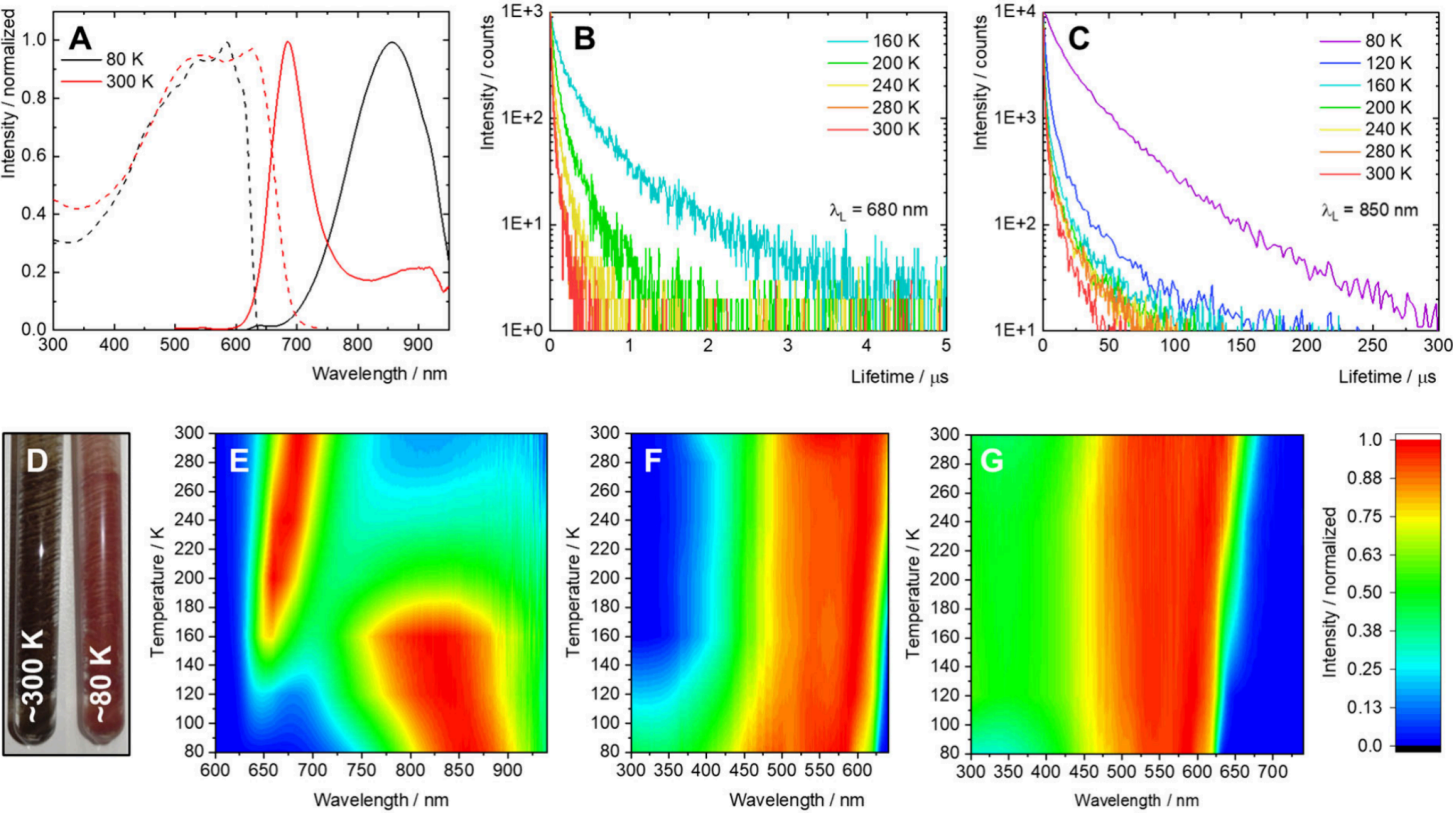
(A) Normalized photoluminescence spectra of solid **nMo**_**6**_ **I**_**12**_ at 80 and 300 K, excited at 450 nm, and corresponding excitation spectra recorded at 850 nm. (B) Luminescence decay kinetics of solid **nMo**_**6**_ **I**_**12**_ from 160 to 300 K, excited at 405 nm, recorded at 680 nm. (C) Luminescence decay kinetics of solid **nMo**_**6**_ **I**_**12**_ from 80 to 300 K, excited at 405 nm, recorded at 850 nm. (D) Photographs of solid **nMo**_**6**_ **I**_**12**_ at ∼80 and ∼300 K. (E) Contour plot of the normalized emission spectra from 80 to 300 K and corresponding contour plots of normalized excitation spectra from 80 to 300 K, recorded at (F) 680 and (G) 850 nm.

The red emission can be tentatively attributed to the radiative recombination of photogenerated electrons in the conduction band of **nMo**_**6**_ **I**_**12**_ with holes in the valence band. The significant blue-shit of the excitation band with a decrease in temperature and the low photoluminescence quantum yield are consistent with the behavior of an indirect-band gap semiconductor, where light absorption and radiative recombination are mediated by phonons. The NIR emission can be tentatively attributed to defects such as surface or crystal dislocations. These defects result in electron traps with an energy that is lower than that of the bottom of the conduction band. Excited states associated with electron traps are usually longer-lived than that of electrons in the conduction band, and their radiative relaxation is promoted at low temperatures where vibrational quenching is decreased.^27,28^

Singlet oxygen, O_2_(^1^Δ_g_), produced by [Mo_6_I^i^_8_L^a^_6_]*^n^* molecular complexes was previously evidenced by measuring its weak phosphorescence signal centered at 1274 nm.^4^ Such a signal was not observed for oxygen-saturated water, acetone, or acetonitrile dispersions of **nMo**_**6**_ **I**_**12**_, suggesting that no significant amount of O_2_(^1^Δ_g_) is produced. Still, **nMo**_**6**_ **I**_**12**_ might produce other reactive oxygen species (ROS) via a photocatalytic process involving oxygen/water molecules (superoxide, hydrogen peroxide, and hydroxyl radical). We evaluated the overall formation of ROS using dichlorodihydrofluorescein diacetate (DCF-DA), which is converted into highly green fluorescent 2′,7′-dichlorofluorescein upon reaction with ROS (Figure 8A). Notably, the rate of oxidation of DCF-DA was comparable in the presence and absence of oxygen, suggesting that the formation of ROS includes an oxygen-independent pathway, probably via hole injection to water molecules to form a hydroxyl radical. This hypothesis was validated by monitoring the reaction between coumarin and hydroxyl radicals, which yielded highly fluorescent product 7-hydroxycoumarin (Figure 8B and Figure S7). The results confirmed hydroxyl radical formation, with higher yields observed under anoxic conditions than in normoxia. The reduced yield in the presence of oxygen is attributed to a competing pathway, in which oxygen acts as an electron scavenger. This shift can alter the dominant hydroxyl radical-driven hydroxylation into a slower pathway mediated by superoxide and/or hydrogen peroxide. Consequently, in this specific reaction, the absence of oxygen enables the most efficient oxidative route to proceed unimpeded.

**Figure 8:**
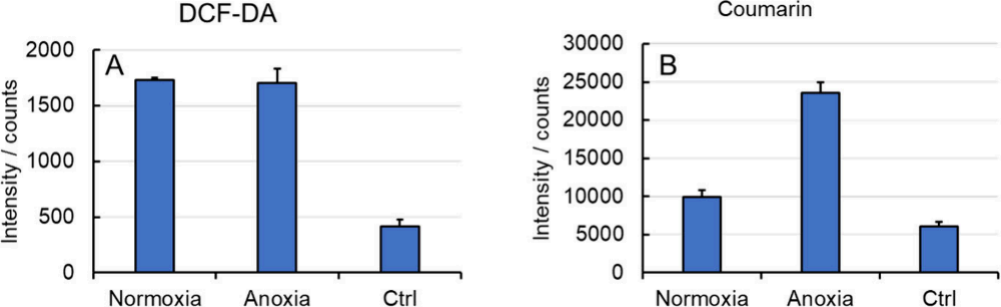
(A) Formation of ROS in an aqueous dispersion of **nMo**_**6**_ **I**_**12**_ irradiated at 460 nm in the presence of 10 μM DCF-DA as an oxidative probe. The ordinate corresponds to the fluorescence intensity of 2′,7′-dichlorofluorescein, the oxidized form of DCF-DA, at 525 nm, upon excitation at 488 nm. The fluorescence intensity was recorded after illumination for 5 min under normoxia and anoxia. (B) Formation of hydroxyl radicals in an aqueous dispersion of **nMo**_**6**_ **I**_**12**_ irradiated at 460 nm in the presence of 50 μM coumarin as a hydroxyl radical probe. The ordinate corresponds to the fluorescence intensity of 7-hydroxycoumarin, the hydroxylated form of coumarin, at 455 nm, upon excitation at 343 nm. The fluorescence intensity was recorded after illumination for 2 h under normoxia and anoxia. Controls (Ctrl) were recorded under the same conditions in the absence of **nMo**_**6**_ **I**_**12**_.

### Electronic Structure

Ligated octahedral cluster compounds have been extensively investigated in the literature using first-principles calculations. The electronic structure of many of these compounds is in turn governed by the octahedral metallic units and can be rationalized on the basis of the molecular orbital (MO) diagram of the isolated units, whether the bonded ligands cap the metal–metal bonds (M_6_L^i^_12_L^a^_6_) or the triangular metallic faces (M_6_L^i^_8_L^a^_6_) (M is a transition metal, and L is a two-electron donor ligand).^10,11^ The electronic structure of Mo_6_I_12_ follows this trend. Previous theoretical studies showed that the MO diagram of an isolated [Mo_6_I^i^_8_I^a^_6_]^*n*−^ unit exhibits a large HOMO–LUMO gap separating the bonding Mo–Mo MOs from the antibonding ones for the dianionic isolated units (*n* = 2). This corresponds to an optimal valence electron count of 24 for such a unit. The density of states (DOS) computed for Mo_6_I_12_ is sketched in Figure 9. A large energy gap separates the occupied valence band from the conduction band. This suggests that the interactions between neighboring units via shared apical iodine atoms and between slabs of clusters are much less important than interactions between (among) metal and halogen atoms within the octahedral units. It is noteworthy to mention that all Mo atoms contribute, almost equally, to the bands around the Fermi level. For iodine atoms, I4 atoms contribute the most to the top of the valence band. These ligands are apical ligands oriented to the interlayer space, not shared with neighboring clusters. Iodine atoms that contribute the most to the bottom of the conduction band are inner ligands (I1 and I2 (cf. Figure 9)).

**Figure 9:**
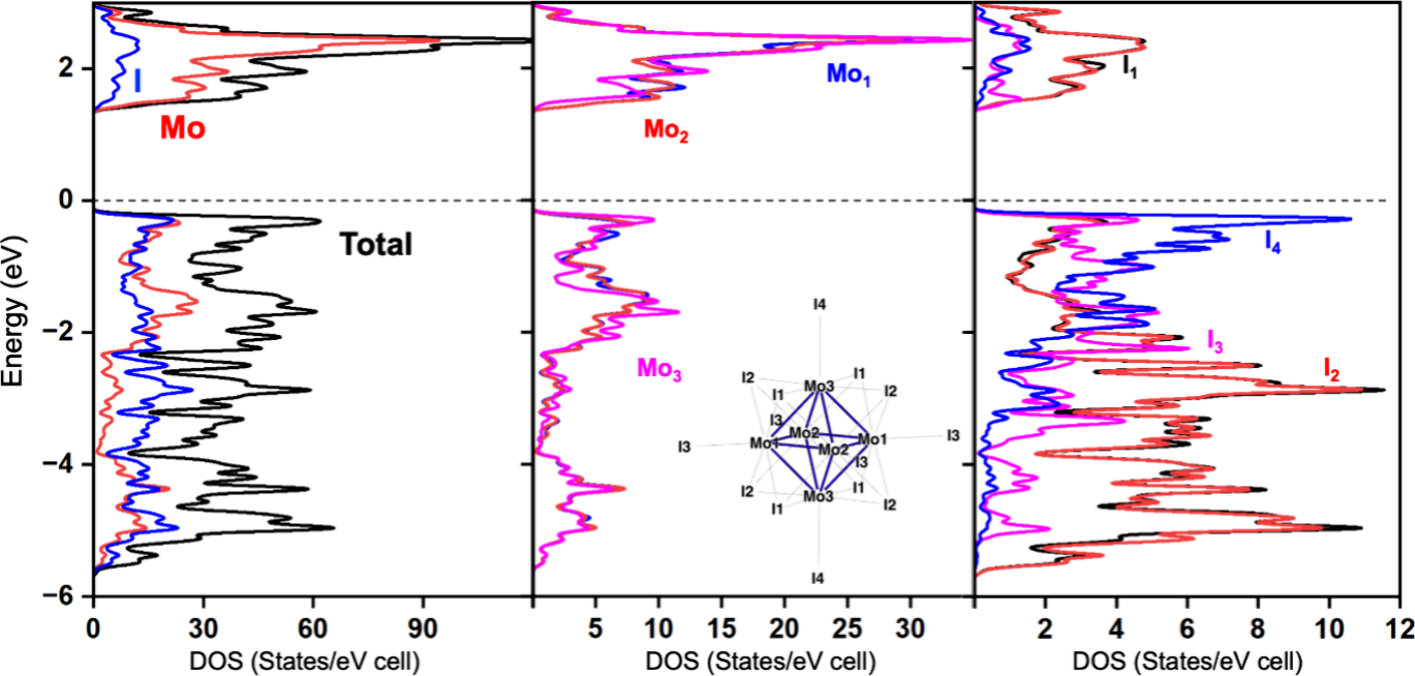
Total and atom-projected DOS values computed for Mo_6_I_12_. The labeling of the respective atoms is depicted in the middle panel.

The band structure of Mo_6_I_12_ is sketched in Figure 10, confirming a band gap of about 1.6 eV. This value, lower than those obtained using the Tauc plots (Figures S4 and S5), is probably underestimated, which is often the case for semiconducting and insulating materials with DFT calculations using LDA and GGA exchange-correlation functionals.^29^ The band structure indicates that the gap is indirect. The top of the valence is located in the Γ → *Z* region, whereas the bottom of the conduction band lies at *Y*. Few additional models were carried out to evaluate the influence of the nanosized compound, i.e., a model compound with one, two, and three slabs of Mo_6_I_12_. Their band structures are sketched in Figure S8. Even if some differences occur between these band structures, the band gap is almost unaffected by the number of Mo_6_I_12_ slabs considered in the model. From a theoretical point of view, it suggests that the nanosizing of the title compound hardly affects its electronic structure and, consequently, its luminescence properties.

**Figure 10:**
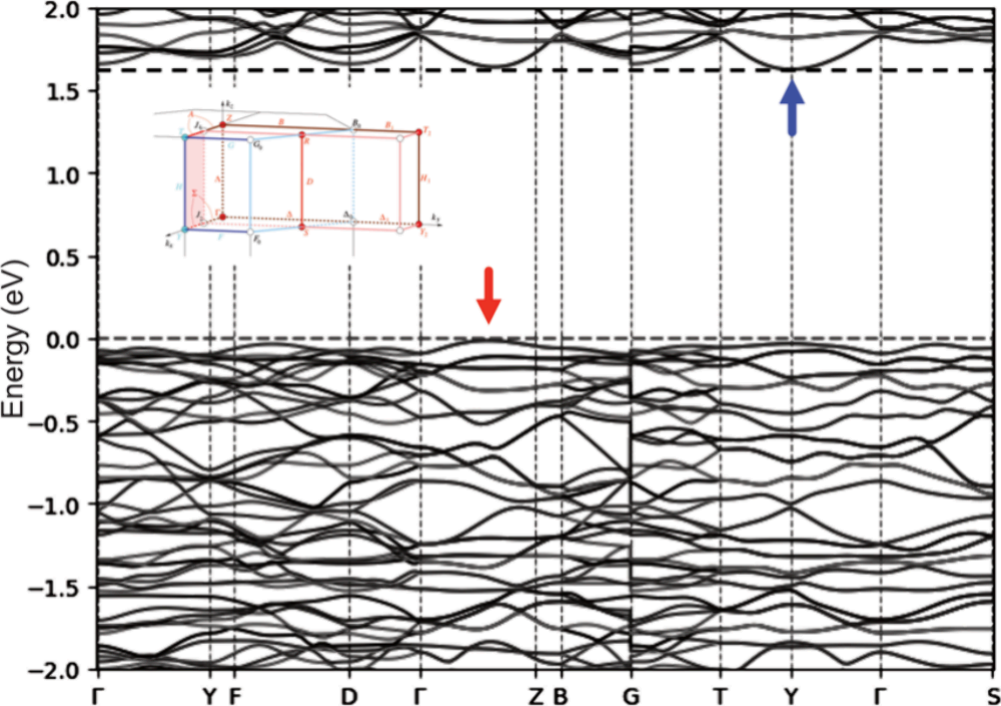
Band structure computed for Mo_6_I_12_. The top of the valence band and the bottom of the conduction band are shown with red and blue arrows, respectively.

### Photodynamic Inactivation of Bacteria

In the context of water disinfection, photocatalytic inactivation of pathogens is advantageous as it efficiently eliminates harmful microorganisms without generating toxic byproducts, making it a safe and sustainable treatment method. The photogenerated ROS demonstrated for an aqueous dispersion of **nMo**_**6**_ **I**_**12**_ prompted us to investigate its potential for antimicrobial photodynamic inactivation using several strains in the form of planktonic cultures, namely, Gram-positive *E. faecalis* and *S. aureus* and Gram-negative *E. coli* (Figure 11). In the dark, nanocrystals of **nMo**_**6**_ **I**_**12**_ were nontoxic against all bacterial strains in the tested concentration range. After irradiation with a blue LED light (460 nm, 15 min), notable photoinactivation was observed for Gram-positive *E. faecalis* and *S. aureus* with a decrease in the relative viability by 99.992% and 99.540%, respectively, at 70 μg mL^–1^ **nMo**_**6**_ **I**_**12**_. On the other hand, no photoinactivation was observed for Gram-negative *E. coli*, which is consistent with the higher resistance of Gram-negative strains due to the more complex constitution of the cell wall and membrane.

**Figure 11:**
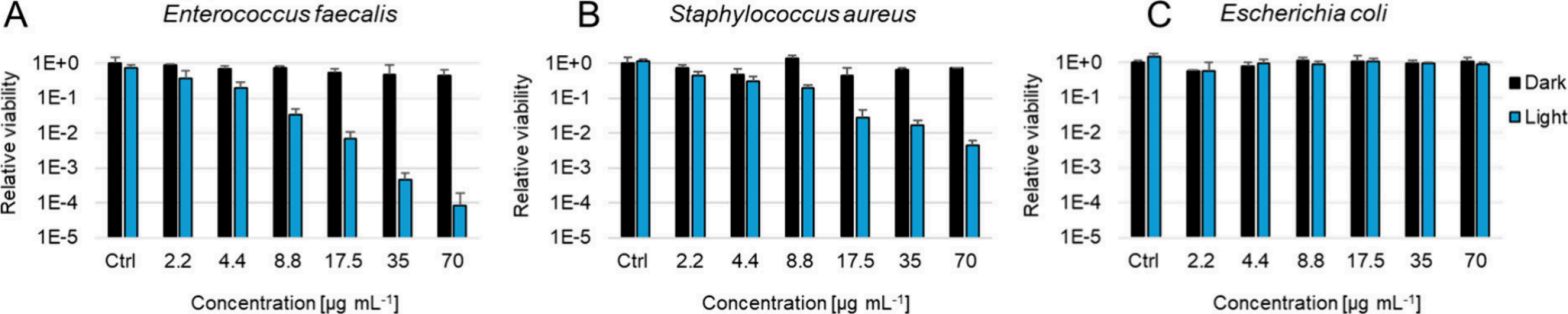
Dark toxicity and phototoxicity toward planktonic cultures of (A) *E. faecalis*, (B) *S. aureus*, and (C) *E. coli* in the range of 2.2–70 μg mL^–1^ **nMo**_**6**_ **I**_**12**_. Incubation for 2 h and then irradiation at 460 nm for 15 min (18 mW cm^–2^). The controls were performed in the absence of **nMo**_**6**_ **I**_**12**_.

To assess the biosafety and selectivity of the nanoparticles for potential water disinfection applications, their effects on mammalian cells were investigated alongside their known antibacterial photoinactivation activity. The cytotoxicity of **nMo**_**6**_ **I**_**12**_ in the dark and under blue-light illumination was evaluated on HeLa cells incubated in the absence and presence of fetal bovine serum (FBS). Under all conditions, the nanoparticles showed little to no cytotoxic effect on HeLa cells cultured in complete medium, with blue-light exposure not inducing significant additional loss of viability compared to dark controls (Figure 12). The presence of FBS during incubation slightly decreased the already minimal effect, likely due to a reduced cellular uptake. In contrast, **nMo**_**6**_ **I**_**12**_ efficiently photoinactivated bacteria in pure water under blue light, highlighting a strong selectivity between microbial targets in aqueous environments and mammalian cells in nutrient-rich culture media. This discrepancy highlights the favorable safety profile of **nMo**_**6**_ **I**_**12**_ for water disinfection applications, as they exhibit potent antibacterial activity under operational conditions while remaining largely inactive and nontoxic toward human cells, even upon light activation.

**Figure 12:**
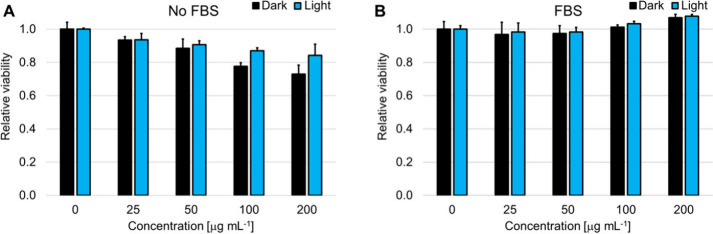
Dark toxicity and phototoxicity toward HeLa cells in 25–200 μg mL^–1^ **nMo**_**6**_ **I**_**12**_. Incubation for 2 h without FBS or with 5% FBS in culture medium and then irradiation at 460 nm for 15 min (18 mW cm^–2^). The controls were performed in the absence of **nMo**_**6**_ **I**_**12**_.

## Conclusion

We have demonstrated here the successful top-down preparation of nanocrystalline molybdenum­(II) iodide using ultrasonication, preserving the crystallographic and chemical integrity of the original bulk material. Detailed characterization by XRD, Raman spectroscopy, electron microscopy, XPS, and DFT calculations confirmed the structural and electronic properties of the nanocrystals, revealing an indirect band gap and photoluminescent behavior consistent with defect-mediated emission in the NIR region. Notably, despite a low luminescence quantum yield at room temperature, the defect-mediated emission in the NIR region is significantly enhanced at cryogenic temperatures, indicating strong nonradiative relaxation pathways under ambient conditions.

Our photophysical investigations indicated that **nMo**_**6**_ **I**_**12**_ does not generate O_2_(^1^Δ_g_) under 460 nm light but instead produces ROS, particularly hydroxyl radicals, predominantly through oxygen-independent mechanisms. This unique ROS generation profile substantiates the observed potent antibacterial activity of **nMo**_**6**_ **I**_**12**_ under 460 nm light against Gram-positive bacterial strains. No toxicity was observed in the dark, and negligible activity was detected against *E. coli*, consistent with the structural resistance mechanisms of Gram-negative bacteria.

The low toxicity of **nMo**_**6**_ **I**_**12**_ on HeLa cells highlighted the favorable safety profile for the water disinfection application. Taken as a whole, these findings establish nanocrystalline **nMo**_**6**_ **I**_**12**_ as a promising photodynamic antimicrobial agent, especially for applications targeting Gram-positive pathogens. The material’s photostability, light-triggered ROS generation, and bactericidal action position it as a valuable candidate for further development in antibacterial surface coatings, wound care, or other light-assisted therapeutic applications. Future work may focus on enhancing its luminescence efficiency and expanding its action spectrum through chemical modifications or nanocomposite strategies.

## Experimental Section

### Reagents and General Procedures

Mo_6_I_12_ was prepared according to a previously published procedure.^30^ Molybdenum and iodine were obtained from Sigma-Aldrich and used as received. Solvents for the synthesis were purchased from Penta (Czech Republic) and dried over molecular sieves (3 Å).

### Preparation of Mo_6_I_12_ Nanocrystals (**nMo**_**6**_ **I**_**12**_)

A 500 mg portion of Mo_6_I_12_ was dispersed in 100 mL of *N*-methyl-2-pyrrolidone (NMP) and then submitted to a high-intensity cavitation field in a pressurized ultrasound reactor for 2 h (UIP2000 hd, 20 kHz, 2000 W, Hielscher Ultrasonics, GmbH, Teltow, Germany). The resulting suspension was centrifuged at 11 000 rpm for 20 min, and the supernatant was discarded. In order to remove traces of NMP and reaction byproducts, the sediment was then submitted to three cycles of redispersion in acetone with the help of an ultrasound bath for 30 min and centrifugation at 11 000 rpm for 5 min. Then, the sediment was redispersed in 50 mL of acetone in an ultrasonic bath for 30 min, and the nanocrystals were isolated by centrifugation of the dispersion at 4000 rpm for 1 min and separation of the supernatant. This cycle was repeated three times, providing 78 mg of **nMo**_**6**_ **I**_**12**_.

### Instrumental Techniques

XPS measurements were performed in an ESCALAB Xi+ X-ray photoelectron spectrometer (ThermoScientific, USA) employing a monochromated Al Kα X-ray source (*h*υ = 1486.6 eV). The C­(1s) level (284.9 eV) was taken as a reference binding energy. High-resolution spectra were collected using an analysis area of 650 μm × 650 μm and a 20 eV pass energy. The charge neutralizer was used for data collection, being monitored using the C­(1s) signal corresponding to adventitious carbon. All spectra were collected and fitted using the Avantage software (ThermoScientific, USA), and a smart background was used for all spectra. Raman measurements were recorded with a Renishaw spectrometer operating at 514 nm. Images of the nanocrystals were acquired by HRTEM FEI Talos F200X (ThermoScientific, USA) and HRSEM FEI NanoSEM 450 (ThermoScientific, USA) instruments. Size distributions and corresponding ζ potentials were determined by dynamic light scattering (DLS) on a particle size analyzer Zetasizer Nano ZS (Malvern, UK). Powder X-ray diffraction (XRD) patterns were recorded using a PANalytical X’Pert PRO diffractometer in the transmission setup equipped with a conventional Cu X-ray tube (40 kV, 30 mA). Luminescence properties were measured on an FLS1000 spectrometer (Edinburgh Instruments, UK) using a cooled PMT-900 photon detection module (Edinburgh Instruments, UK). Aqueous dispersions (0.1 mg mL^–1^ **nMo**_**6**_ **I**_**12**_) were saturated with air or argon to ensure different oxygen concentrations for phosphorescence analyses. The FLS1000 spectrometer was also used for time-resolved phosphorescence measurements (λ_exc_ = 405 nm; VPLED Series), and the recorded decay curves were fitted to exponential functions by Fluoracle version 2.13.2 (Edinburgh Instruments, UK). UV–vis absorption spectra and phosphorescence quantum yields of the samples were recorded using a Quantaurus QY C11347-1 spectrometer (Hamamatsu, Japan).

Evaluation of the photoinduced oxidation of dichlorodihydrofluorescein diacetate (DCF-DA) was performed by adding 5 μL of DCF-DA to 500 μL of air-saturated or deoxygenated **nMo**_**6**_ **I**_**12**_ deionized water dispersions (0.03 mg mL^–1^) in an Eppendorf tube, irradiated with a 12 × 10 W LED source (Cameo, Germany) (460 nm, 18 mW cm^–2^) for 5 min. The solid was centrifuged out to eliminate possible interference, and the fluorescence intensity of the supernatant was measured at wavelengths of 488 and 525 nm (excitation and emission, respectively). Hydroxyl radical generation was detected via its reaction with coumarin, which produces fluorescent 7-hydroxycoumarim. Air-saturated or deoxygenated dispersions of **nMo**_**6**_ **I**_**12**_ (0.03 mg mL^–1^) in deionized water containing 50 μM coumarin were irradiated for 2 h with the 460 nm LED source, and **nMo**_**6**_ **I**_**12**_ was removed by centrifugation (10 000 rpm/5 min). The fluorescence intensities of the solutions were measured at 455 nm (excitation at 343 nm). Deionized water with 50 μM coumarin served as a control.

### Photoinactivation of Bacteria

Bacterial samples of *S. aureus*, *E. faecalis*, and *E. coli* were cultivated at 37 °C and stored at 4 °C on LB agar. The stock inocula of *S. aureus*, *E. faecalis*, and *E. coli* were prepared by diluting bacteria in water and standardizing the suspension to 1 McF. A 100 μL aliquot of the inoculum was taken and mixed with various concentrations of **nMo**_**6**_ **I**_**12**_ (2.2–70 μg mL^–1^). The samples were incubated for 2 h in the dark at laboratory temperature and afterward irradiated with a 12 × 10 W LED source (Cameo, Germany) (460 nm, 18 mW cm^–2^, 15 min). For quantification of inactivated bacteria, the Miles and Misra method on LB agar was used. All experiments were performed in biological triplicate.

### Efficiency against Human Cells

Human cervical adenocarcinoma HeLa cells were maintained in Eagle’s minimum essential medium (EMEM) containing 5% fetal bovine serum (FBS) and 0.5 mM l-glutamine under humidified conditions at 37 °C with 5% CO_2_. Experiments were initiated after cell seeding. After 24 h, the culture medium was exchanged for fresh phenol red-free medium, either supplemented with 5% FBS (complete medium) or lacking FBS, according to the experimental setup. The **nMo**_**6**_ **I**_**12**_ were added to the cultures at defined concentrations; the final acetone content did not exceed 1% (v/v). Following a 2 h incubation with **nMo**_**6**_ **I**_**12**_, cells were either exposed to blue light (460 nm, 15 min, 18 mW cm^–2^) or maintained in the dark. The cell viability was assessed the following day using a resazurin-based assay with fluorescence recorded at excitation and emission wavelengths of 560 and 590 nm, respectively. Control samples were treated identically but without addition of **nMo**_**6**_ **I**_**12**_.

### Computational Details

DFT calculations were carried out for geometry optimizations and band-structure calculations using the VASP code (version 6.2.0).^31^ The generalized gradient approximation (GGA) was used to describe the exchange-correlation (XC) interaction, and the parametrization of Perdew, Burke, and Ernzerhof (PBE) was employed.^32^ van der Waals (VdW) corrections were considered in the computation process using the DFT-D3 method as proposed by Grimme.^33^ Projector-augmented wave potentials were used for all atoms.^34^ Calculations were performed using a cutoff energy of 450 eV. The Monkhorst–Pack method was used to sample the irreducible Brillouin zone for the calculations of the electronic wave functions.^35^ Symmetry constraints were considered for the optimization of the cell parameters and atomic positions. Optimized bond distances compare very well with that resulting from X-ray diffraction studies (see Table S5).^36^

Raman calculations were performed with the Crystal 23 program on the VASP geometry-optimized crystal structure of Mo_6_I_12_.^37^ Raman intensities were evaluated analytically, through a coupled-perturbed-Hartree–Fock/Kohn–Sham (CPHF/KS) scheme.^23,24^ The PBE exchange-correlation functional was considered.^32^ A triple-ζ valence + polarization basis set of Gaussian-type functions including a pseudopotential was adopted for Mo and I atoms according to the Peintinger–Oliveira–Bredow method.^38,39^ Reciprocal space was sampled with 21 k-points in the irreducible part of the Brillouin zone.

## Supplementary Material



## Notes

Additional experimental data, including X-ray diffraction, Raman spectroscopy, X-ray photoelectron spectroscopy, and dynamic light scattering data, photoluminescence measurements, and DFT calculations (PDF)

## References

[1] Schafer H., von Schnering H. G., Tillack J., Kuhnen F., Wohrle H., Baumann H. (1967). Neue Untersuchungen über die Chloride des Molybdäns. Z. Anorg. Allg. Chem..

[2] Brauer, G. ; Handbuch der Präparativen Anorganischen Chemie. 3., umgearbeitete Auflage. Band III; Ferdinand Enke: Stuttgart, Germany, 1981.

[3] Maverick A. W., Najdzionek J. S., MacKenzie D., Nocera D. G., Gray H. B. (1983). Spectroscopic,
Electrochemical, and Photochemical Properties of Molybdenum­(II) and
Tungsten­(II) Halide Clusters. J. Am. Chem. Soc..

[4] Kirakci K., Kubát P., Langmaier J., Polívka T., Fuciman M., Fejfarová K., Lang K. (2013). A Comparative Study
of the Redox and Excited State Properties of (nBu_4_N)_2_[Mo_6_X_14_] and (nBu_4_N)_2_[Mo_6_X_8_(CF_3_COO)_6_] (X = Cl, Br, or I). Dalton Trans..

[5] Khlifi S., Taupier G., Amela-Cortes M., Dumait N., Freslon S., Cordier S., Molard Y. (2021). Expanding
the Toolbox of Octahedral
Molybdenum Clusters and Nanocomposites Made Thereof: Evidence of Two-Photon
Absorption Induced NIR Emission and Singlet Oxygen Production. Inorg. Chem..

[6] Kirakci K., Kubát P., Fejfarová K., Martinčík J., Nikl M., Lang K. (2016). X-Ray Inducible
Luminescence and
Singlet Oxygen Sensitization by an Octahedral Molybdenum Cluster Compound:
A New Class of Nanoscintillators. Inorg. Chem..

[7] Feliz M., Atienzar P., Amela-Cortés M., Dumait N., Lemoine P., Molard Y., Cordier S. (2019). Supramolecular
Anchoring of Octahedral
Molybdenum Clusters onto Graphene and Their Synergies in Photocatalytic
Water Reduction. Inorg. Chem..

[8] Kirakci K., Zelenka J., Rumlová M., Martinčík J., Nikl M., Ruml T., Lang K. (2018). Octahedral molybdenum
clusters as radiosensitizers for X-ray induced photodynamic therapy. J. Mater. Chem. B.

[9] Aliev Z. G., Klinkova L. A., Dubrovin I. V., Atovmyan L. O. (1981). Preparation and
Structure of Molybdenum Di-iodide. Zh. Neorg.
Khim..

[10] Hughbanks T., Hoffmann R. (1983). Molybdenum chalcogenides: clusters, chains, and extended
solids. The Approach to Bonding in Three Dimensions. J. Am. Chem. Soc..

[11] Hughbanks T. (1989). Bonding in
Clusters and Condensed Cluster Compounds that Extend in One, Two and
Three Dimensions. Prog. Solid State Chem..

[12] Zhang M., Wang K., Zeng S., Xu Y., Nie W., Chen P., Zhou Y. (2021). Visible Light-Induced
Antibacterial
Effect of MoS2: Effect of the Synthesis Methods. Chem. Eng. J..

[13] Guégan R., Cheng X., Huang X., Němečková Z., Kubáňová M., Zelenka J., Ruml T., Grasset F., Sugahara Y., Lang K., Kirakci K. (2023). Graphene Oxide
Sheets Decorated with Octahedral Molybdenum Cluster Complexes for
Enhanced Photoinactivation of Staphylococcus Aureus. Inorg. Chem..

[14] Novikova E. D., Pronina E. V., Vorotnikov Y. A., Adamenko L. S., Alekseev A. Y., Shestopalov A. M., Tsygankova A. R., Gusel’nikova T.
Ya, Kubát P., Kirakci K., Lang K., Shestopalov M. A. (2023). Cotton
Fabrics Modified with Molybdenum Nanoclusters for Photodynamic Inactivation
of Bacteria and Viruses. J. Environ. Chem. Eng..

[15] Kirakci K., Zelenka J., Rumlová M., Cvačka J., Ruml T., Lang K. (2019). Cationic Octahedral
Molybdenum Cluster
Complexes Functionalized with Mitochondria-Targeting Ligands: Photodynamic
Anticancer and Antibacterial Activities. Biomater.
Sci..

[16] Kirakci K., Kubáňová M., Přibyl T., Rumlová M., Zelenka J., Ruml T., Lang K. (2022). A Cell Membrane
Targeting Molybdenum-Iodine Nanocluster: Rational Ligand Design toward
Enhanced Photodynamic Activity. Inorg. Chem..

[17] Kirakci K., Nguyen T. K. N., Grasset F., Uchikoshi T., Zelenka J., Kubát P., Ruml T., Lang K. (2020). Electrophoretically
Deposited Layers of Octahedral Molybdenum Cluster Complex: A Promising
Coating for Mitigation of Pathogenic Bacterial Biofilms under Blue
Light. ACS Appl. Mater. Interfaces.

[18] Beltrán A., Mikhailov M., Sokolov M. N., Pérez-Laguna V., Rezusta A., Revillo M. J., Galindo F. (2016). A photobleaching resistant
polymer supported hexanuclear molybdenum iodide cluster for photocatalytic
oxygenations and photodynamic inactivation of Staphylococcus aureus. J. Mater. Chem. B.

[19] Štengl V., Henych J. (2013). Strongly Luminescent
Monolayered MoS_2_ Prepared
by Effective Ultrasound Exfoliation. Nanoscale.

[20] Kumar D., Katiyar N., Kumar P., Sharma P., Sharma S. K. (2021). Ultrasonication:
An Intensifying Tool for Preparation of Stable Nanofluids. J. Mol. Liq..

[21] Jiang L.-P., Xu S., Zhu J.-M., Zhang J.-R., Zhu J.-J., Chen H.-Y. (2004). Ultrasonic-Assisted
Synthesis of Monodisperse Single-Crystalline Silver Nanoplates and
Gold Nanorings. Inorg. Chem..

[22] Bhangu S. K., Baral A., Zhu H., Ashokkumar M., Cavalieri F. (2021). Sound Methods for the Synthesis of
Nanoparticles from
Biological Molecules. Nanoscale Adv..

[23] Maschio L., Kirtman B., Rérat M., Orlando R., Dovesi R. (2013). Ab Initio
Analytical Raman Intensities for Periodic Systems Through a Coupled
Perturbed Hartree–Fock/Kohn–Sham Method in an Atomic
Orbital Basis. I. Theory. J. Chem. Phys..

[24] Maschio L., Kirtman B., Rérat M., Orlando R., Dovesi R. (2013). Ab initio
Analytical Raman Intensities for Periodic Systems Through a Coupled
Perturbed Hartree–Fock/Kohn–Sham Method in an Atomic
Orbital Basis. II. Validation and Comparison with Experiments. J. Chem. Phys..

[25] Schoonover J. R., Zietlow T. C., Clark D. L., Heppert J. A., Chisholm M. H., Gray H. B., Sattelberger A. P., Woodruff W. H. (1996). Resonance Raman
Spectra of [M_6_X_8_Y_6_]^2‑^ Cluster Complexes (M = Mo, W; X, Y = Cl, Br, I). Inorg. Chem..

[26] Kirakci K., Zelenka J., Rumlová M., Martinčík J., Nikl M., Ruml T., Lang K. (2018). Octahedral Molybdenum
Clusters as Radiosensitizers for X-Ray Induced Photodynamic Therapy. J. Mater. Chem. B.

[27] van
Dijken A., Meulenkamp E. A., Vanmaekelbergh D., Meijerink A. (2000). The Kinetics of the Radiative and Nonradiative Processes
in Nanocrystalline ZnO Particles upon Photoexcitation. J. Phys. Chem. B.

[28] Blumenau A. T., Jones R., Öberg S., Briddon P. R., Frauenheim T. (2001). Dislocation
Related Photoluminescence in Silicon. Phys.
Rev. Lett..

[29] Perdew J. P., Zunger A. (1981). Self-Interaction Correction to Density-Functional Approximations
for Many-Electron Systems. Phys. Rev. B.

[30] Kirakci K., Cordier S., Perrin C. (2005). Synthesis
and Characterization of
Cs_2_Mo_6_X_14_ (X = Br or I) Hexamolybdenum
Cluster Halides: Efficient Mo_6_ Cluster Precursors for Solution
Chemistry Syntheses. Z. Anorg. Allg. Chem..

[31] Kresse G., Furthmüller J. (1996). Efficiency
of Ab-Initio Total Energy Calculations for Metals and Semiconductors
Using a Plane-Wave Basis Set. Comput. Mater.
Sci..

[32] Perdew J. P., Burke K., Ernzerhof M. (1996). Generalized
Gradient Approximation
Made Simple. Phys. Rev. Lett..

[33] Grimme S., Antony J., Ehrlich S., Krieg S. (2010). A Consistent and Accurate
Ab Initio Parametrization of Density Functional Dispersion Correction
(DFT-D) for the 94 Elements H-Pu. J. Chem. Phys..

[34] Blöchl P. E. (1994). Projector Augmented-Wave
Method. Phys. Rev. B.

[35] Monkhorst H. J., Pack J. D. (1976). Special Points for Brillouin-Zone
Integrations. Phys. Rev. B.

[36] Aliev Z. G., Klinkova L. A., Dubrovin I. V., Atovmyan L. O. (1981). Preparation and
Structure of Molybdenum Di-Iodide. Zh. Neorgan.
Khimii.

[37] Erba A., Desmarais J. K., Casassa S., Civalleri B., Donà L., Bush I. J., Searle B., Maschio L., Edith-Daga L., Cossard A., Ribaldone C., Ascrizzi E., Marana N. L., Flament J.-P., Kirtman B. (2023). CRYSTAL23:
A Program for Computational Solid State Physics and Chemistry. J. Chem. Theory Comput..

[38] Vilela
Oliveira D., Laun J., Peintinger M. F., Bredow T. (2019). BSSE-Correction Scheme for Consistent Gaussian Basis
Sets of Double- and Triple-Zeta Valence with Polarization Quality
for Solid-State Calculations. J. Comput. Chem..

[39] Laun J., Bredow T. (2022). BSSE-Corrected Consistent
Gaussian Basis Sets of Triple-Zeta
Valence with Polarization Quality of the Fifth Period for Solid-State
Calculations. J. Comput. Chem..

