# Supplementary material for: Downsizing Mo6I12 to Nanocrystals Unveils Visible-Light Photocatalytic Antibacterial Activity
Source: Inorg Chem. 2026 Mar 18;65(12):6621–30. doi: 10.1021/acs.inorgchem.5c05876 (PMC13040533; doi:10.1021/acs.inorgchem.5c05876)
Supplement: Supplementary file 1 [file ic5c05876_si_001.pdf]

Supporting Information

Downsizing Mo_6I_{12} to Nanocrystals Unveils Visible-Light Photocatalytic Antibacterial Activity.

Michaela Kubáňová[1,2], Martin Šťastný[2], Eric Bourhis[3], Petr Bezdička[2], Jakub Tolasz[2], Aimin Yao,[4] Jean-François Halet,[4,5] Mouna Ben Yahia,[6] Régis Gautier,[4] Jaroslav Zelenka[1], Kamil Lang[2], Régis Guégan[3], Kaplan Kirakci[2]*

[1]*Department of Biochemistry and Microbiology, University of Chemistry and Technology Prague, 166 28 Praha, Czech Republic*

[2]*Institute of Inorganic Chemistry of the Czech Academy of Sciences, 250 68 Husinec-Řež, Czech Republic*

[3]*Interfaces, Confinement, Matériaux et Nanostructures ICMN-UMR 7374, CNRS-Université d'Orléans, 1 Rue de la Férollerie, 45100 Orléans, France*

[4]*Univ Rennes, École Nationale Supérieure de Chimie de Rennes, CNRS, ISCR UMR 6226, 35000 Rennes, France*

[5]*CNRS – Saint-Gobain – NIMS, IRL 3629, Laboratory for Innovative Key Materials and Structures (LINK), 1-1 Namiki, Tsukuba 305-0044, Japan.*

[6]*Institute Charles Gerhardt of Montpellier, Univ. Montpellier, CNRS, ENSCM, Montpellier, France*

Corresponding author: Kaplan Kirakci, kaplan@iic.cas.cz

Content

Figure S1. Powder X-ray diffraction pattern of bulk Mo_6I_{12} with the corresponding Rietveld fit.

Table S1. Structural parameters of bulk Mo_6I_{12} and **nMo_6I_{12}** obtained by the Rietveld refinement.

Table S2. Wavenumbers (in cm^{-1}) of the most intense Raman modes computed for Mo_6I_{12}. Their symmetry and intensity (in arbitrary unit) are given in parentheses.

Figure S2. XPS spectra and corresponding fits of C 1*s* and O 1*s* core levels signals observed in the **nMo_6I_{12}** sample.

Table S3. Analysis of the XPS spectra of **nMo_6I_{12}** sample.

Figure S3. Size distribution by intensity of acetone and deionized water dispersions of **nMo_6I_{12}**, as obtained by dynamic light scattering.

Table S4. Mean size by number, Z-average and polydispersity index of acetone and deionized water dispersions of **nMo_6I_{12}**, as obtained by dynamic light scattering. Zeta potential of aqueous dispersion of **nMo_6I_{12}**, as obtained by electrophoretic light scattering.

Figure S4. Normalized absorption spectrum and excitation spectra of water dispersion and solid sample of **nMo_6I_{12}**, recorded at 680 and 850 nm.

Figure S5. Tauc plots derived from absorption spectra of water dispersion and solid **nMo_6I_{12}**.

Figure S6. Comparison of photoluminescence and radioluminescence of solid **nMo_6I_{12}**.

Figure S7: Details of hydroxyl radical detection.

Figure S8: DFT band structures of model compounds with 1, 2, and 3 slabs of Mo_6I_{12}.

Table S5. Main bond distances resulting from X-ray diffraction studies and DFT calculations. The atomic labels are identical to the one given in Figure 9.

Figure S1. Powder X-ray diffraction pattern of bulk Mo_6I_{12} with the corresponding Rietveld fit.

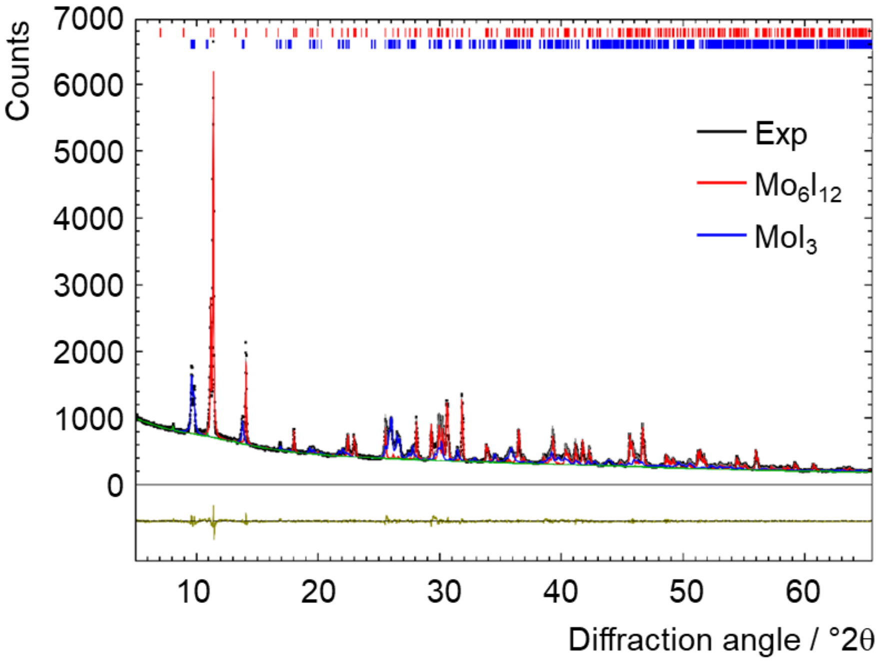

Table S1. Structural parameters of bulk Mo_6I_{12} and **nMo_6I_{12}** obtained by the Rietveld refinement.

Sample	Crystallite size (τ)[a], nm	Lattice constant (*a*), nm	Lattice constant (*b*), nm	Lattice constant (*c*), nm
Mo_6I_{12}	138.2 ± 1.8	1.582793 ± 0.000045	1.255573 ± 0.000043	1.257142 ± 0.000031
**nMo_6I_{12}**	108.0 ± 2.0	1.582128 ± 0.000052	1.256650 ± 0.000035	1.256076 ± 0.000096

[a]τ = K.λ/ β.cos θ (τ : average size of the ordered domains, K : shape factor, λ : X-ray wavelength, β : line broadening at FWHM after subtracting the instrumental line broadening, in radians, θ : Bragg angle)

Table S2. Wavenumbers (in cm^{-1}) of the most intense Raman modes computed for Mo_6I_{12}. Their symmetry and intensity (in arbitrary unit) are given in parentheses.

Frequency (cm^{-1}) (symmetry and intensity)	Assignments
37(B_{2g} 47), 41 (B_{3g} 50)	Mo-$I^{a/a}$
72 (B_{3g} 29), 72 (B_{2g} 36), 77 (B_{3g} 15)	Mo-I^{a}
94 (A_g 13), 101 (A_g 17), 103 (B_{2g} 13), 104 (A_g 15), 104 (B_{3g} 29), 105 (B_{2g} 39), 107 (A_g 21), 107 (B_{1g} 22), 117 (B_{1g} 21), 128 (A_g 39), 134 (A_g 88),<br>135 (B_{2g} 94), 136 (B_{3g} 111), 139 (B_{2g} 33), 139 (B_{3g} 46), 139 (B_{1g} 52)	Mo-Mo and Mo-I^{i}
171 (A_g 1000), 226 (B_{1g} 15), 228 (B_{3g} 12), 230 (A_g 170), 234 (A_g 112), 237 (A_g 118)	Mo-I^{i}
313 (B_{2g} 12), 314 (B_{3g} 18), 315 (A_g 245)	Mo-Mo

Figure S2. XPS spectra and corresponding fits of C 1*s* (**A**) and O 1*s* (**B**) core level signals observed in the **nMo_6I_{12}** sample.

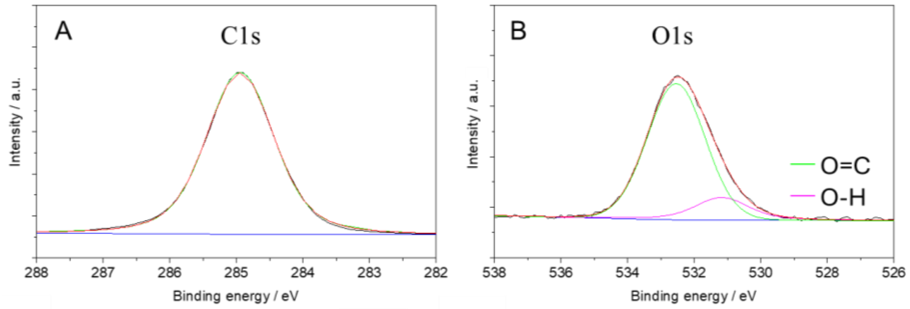

Table S3. Analysis of the XPS spectra of **nMo_6I_{12}** sample.

Core level	Binding energy / eV	FWHM / eV	Area / %
Mo 3$d_{5/2}$	228.9	1.25	53.5
Mo 3$d_{3/2}$	232.0	1.54	46.5
C 1*s*	284.9	1.21	100
O 1*s* (O-H)	531.2	1.94	15.8
O 1*s* (O=C)	532.5	2.05	84.2
I 3$d_{5/2}$ (I^{a})	619.1	1.48	20.7
I 3$d_{5/2}$ (I^{i})	620.5	1.48	39.6
I 3$d_{3/2}$ (I^{a})	630.6	1.45	13.3
I 3$d_{3/2}$ (I^{i})	632.0	1.45	26.3

Figure S3. Size distribution by intensity of acetone (green) and deionized water (red) dispersions of **nMo_6I_{12}**, as obtained by dynamic light scattering.

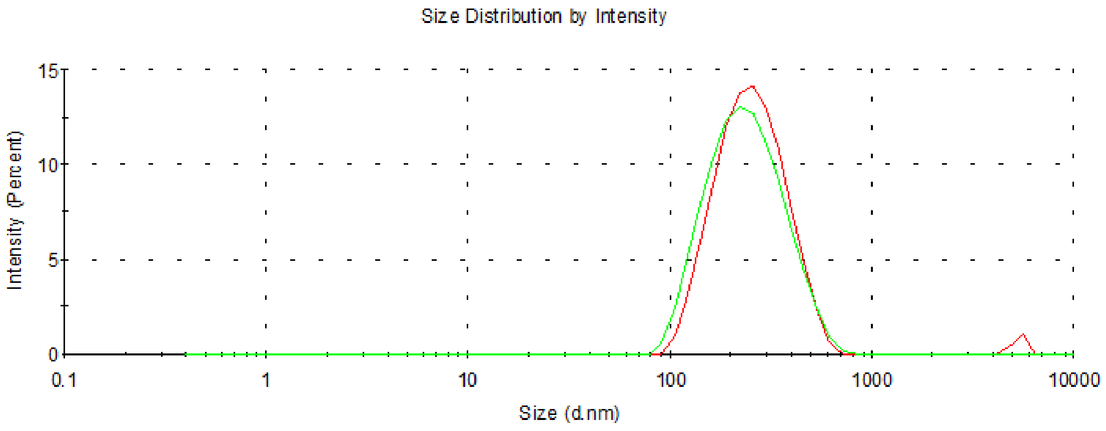

Table S4. Mean size by number, Z-average and polydispersity index (PDI) of acetone and deionized water dispersions of **nMo_6I_{12}**, as obtained by dynamic light scattering. *Zeta* potential of **nMo_6I_{12}** in deionized water (pH~6), as obtained by electrophoretic light scattering.

Solvent	Number mean, d / nm	*Z*-average, d / nm	PDI	ζ-potential / mV
Acetone	155 ± 66	217	0.14	n.a.
Water	178 ± 70	252	0.22	-22 ± 12

Figure S4. Normalized absorption spectra (Abs) and excitation spectra of water dispersion (**A**) and solid sample (**B**) of **nMo$_6$I$_{12}$**, recorded at 680 and 850 nm.

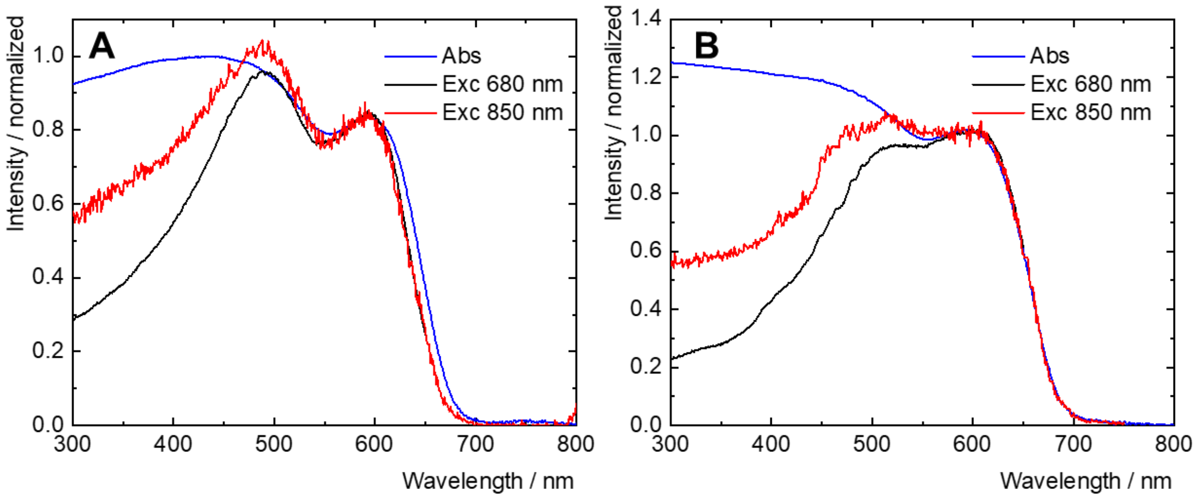

Figure S5. Tauc plots (red) derived from absorption spectra of water dispersion (**A**) and solid sample (**B**) of **nMo$_6$I$_{12}$** at room temperature.

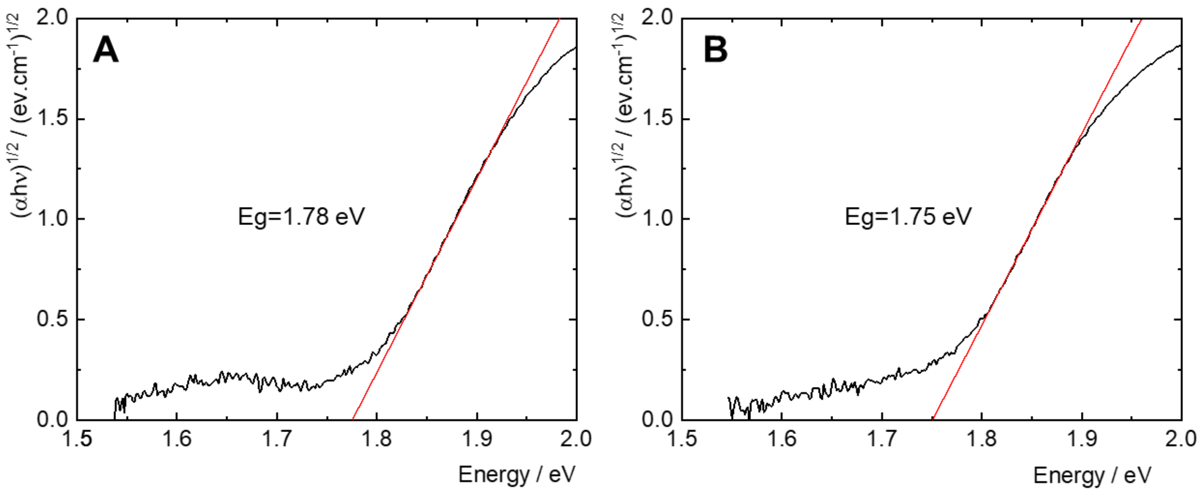

Figure S6. Comparison of photoluminescence (excitation at 450 nm; black) and radioluminescence (60 kV, 200 mA; red) spectra of solid **nMo_6I_{12}**.

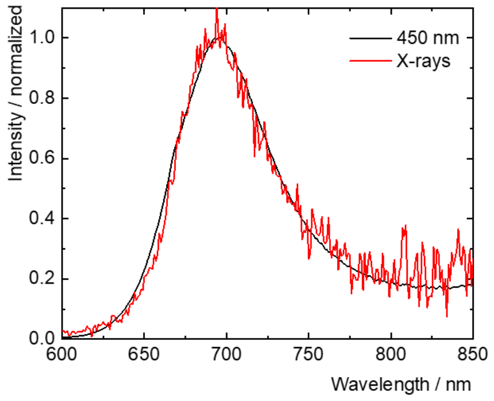

Figure S7: Details of hydroxyl radical detection. Fluorescence spectra of 7-hydroxycoumarin measured at 455 nm after 2 h-irradiation with blue-light of air/Ar-saturated 50 μM coumarin aqueous solutions containing **nMo_6I_{12}** (0.03 mg mL^{-1}). Control experiments (Ctrl) were performed in the absence of **nMo_6I_{12}**.

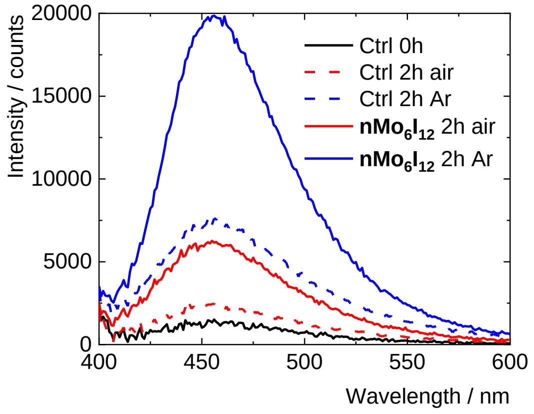

Figure S8: DFT band structures of model compounds with 1, 2, and 3 slabs of Mo_6I_{12}.

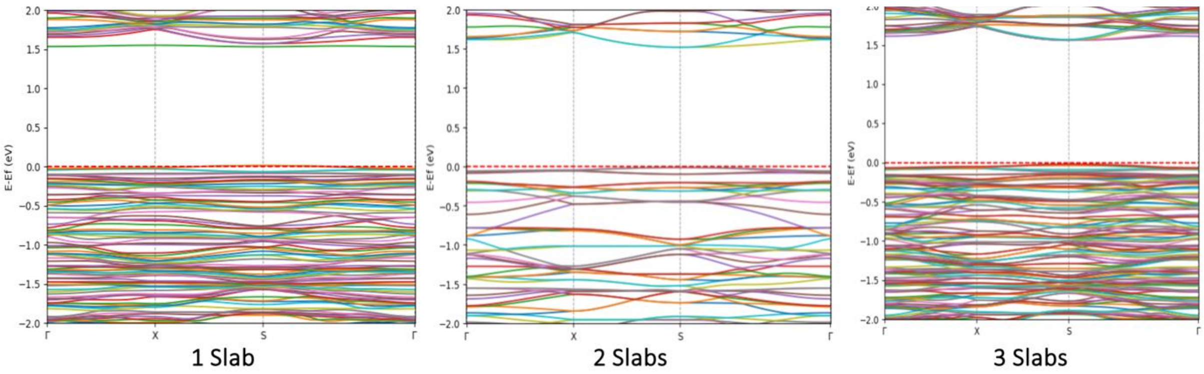

Table S5. Main bond distances resulting from X-ray diffraction studies [1] and DFT calculations. The atomic labels are identical to the one given in Figure 9.

Bonds	X-ray	DFT	Bonds	X-ray	DFT
Mo1-I1	2.6007	2.6186	Mo3-I1	2.5942	2.6149
Mo1-I2	2.6024	2.6167	Mo3-I2	2.5942	2.6124
Mo1-I3	2.5511	2.5599	Mo3-I4	2.6511	2.6664
Mo2-I1	2.5937	2.6086	Mo1-Mo2	2.6300	2.6399
Mo2-I2	2.5955	2.6186	Mo1-Mo3	2.6329	2.6400
Mo2-I3	2.6541	2.6649	Mo2-Mo3	2.6285	2.6324
Mo3-I1	2.5942	2.6149	Mo2-Mo3	2.6310	2.6370

[1] Aliev, Z. G.; Klinkova, L. A.; Dubrovin, I. V.; Atovmyan, L. O., Preparation and Structure of Molybdenum Di-Iodide. *Zh. Neorgan. Khimii* **1981**, *26*, 1964-1967.
